# *StMAPKK1* Enhances Thermotolerance in Potato (*Solanum tuberosum* L.) by Enhancing Antioxidant Defense and Photosynthetic Efficiency Under Heat Stress

**DOI:** 10.3390/plants14152289

**Published:** 2025-07-24

**Authors:** Xi Zhu, Yasir Majeed, Kaitong Wang, Xiaoqin Duan, Nengkang Guan, Junfu Luo, Haifei Zheng, Huafen Zou, Hui Jin, Zhuo Chen, Yu Zhang

**Affiliations:** 1 Key Laboratory of Tropical Fruit Biology, Ministry of Agriculture and Rural Affairs/Key Laboratory of Hainan Province for Postharvest Physiology and Technology of Tropical Horticultural Products, South Subtropical Crops Research Institute, Chinese Academy of Tropical Agricultural Sciences, Zhanjiang 524091, China; zhuxi@catas.cn (X.Z.); gnk7720@163.com (N.G.); ljf10288@163.com (J.L.); zhf20831@163.com (H.Z.); zouhuafen@catas.cn (H.Z.); chenzhuo@catas.cn (Z.C.); 2 National Key Laboratory for Tropical Crop Breeding, Sanya Research Institute, Chinese Academy of Tropical Agricultural Sciences, Sanya 572025, China; 3 State Key Laboratory of Aridland Crop Science, Gansu Agricultural University, Lanzhou 730070, China; kait_wang@163.com (K.W.); dxq2755545382@163.com (X.D.); 4 College of Agronomy, Gansu Agricultural University, Lanzhou 730070, China; 5 College of Tropical Crops, Yunnan Agricultural University, Pu’er 665099, China

**Keywords:** *StMAPKK1*, thermotolerance, *Solanum tuberosum*, antioxidant, photosynthetic efficiency

## Abstract

The functional role of MAPKK genes in potato (*Solanum tuberosum* L.) under high-temperature stress remains unexplored, despite their critical importance in stress signaling and yield protection. We characterized StMAPKK1, a novel group D MAPKK localized to plasma membrane/cytoplasm. Quantitative real-time polymerase chain reaction (qRT-PCR) revealed cultivar-specific upregulation in potato (‘*Atlantic*’ and ‘*Desiree*’) leaves under heat stress (25 °C, 30 °C, and 35 °C). Transgenic lines overexpressing (OE) *StMAPKK1* exhibited elevated antioxidant enzyme activity, including ascorbate peroxidase (APX), catalase (CAT), superoxide dismutase (SOD), and peroxidase (POD), mitigating oxidative damage. Increased proline and chlorophyll accumulation and reduced oxidative stress markers, hydrogen peroxide (H_2_O_2_) and malondialdehyde (MDA), indicate improved cellular redox homeostasis. The upregulation of key antioxidant and heat stress-responsive genes (*StAPX*, *StCAT1/2*, *StPOD12/47*, *StFeSOD2/3*, *StMnSOD*, *StCuZnSOD1/2*, *StHSFA3* and *StHSP20*/*70*/*90*) strengthened the enzymatic defense system, enhanced thermotolerance, and improved photosynthetic efficiency, with significant improvements in net photosynthetic rate (Pn), transpiration rate (E), and stomatal conductance (Gs) under heat stress (35 °C) in *StMAPKK1*-OE plants. Superior growth and biomass (plant height, plant and its root fresh and dry weights, and tuber yield) accumulation, confirming the positive role of *StMAPKK1* in thermotolerance. Conversely, RNA interference (RNAi)-mediated suppression of *StMAPKK1* led to a reduction in enzymatic activity, proline content, and chlorophyll levels, exacerbating oxidative stress. Downregulation of antioxidant-related genes impaired ROS scavenging capacity and declines in photosynthetic efficiency, growth, and biomass, accompanied by elevated H_2_O_2_ and MDA accumulation, highlighting the essential role of *StMAPKK1* in heat stress adaptation. These findings highlight *StMAPKK1*’s potential as a key genetic target for breeding heat-tolerant potato varieties, offering a foundation for improving crop resilience in warming climates.

## 1. Introduction

Potato (*Solanum tuberosum* L.) is one of the world’s most important staple crops, providing a vital source of carbohydrates, vitamins, and minerals for billions of people [1]. Originating in the Andes of South America, potatoes have become a global food security crop due to their high yield, adaptability, and nutritional value [2]. They are the third most consumed crop worldwide after rice and wheat, supporting agricultural economies and food systems in diverse climates [2]. However, potato production faces significant challenges from abiotic stresses, particularly heat stress, which disrupts tuber development, reduces yield, and compromises quality [3]. As global temperatures rise due to climate change, understanding the molecular mechanisms of heat tolerance in potatoes is crucial for breeding resilient varieties [4]. The key pathway involved in stress responses is the mitogen-activated protein kinase (MAPK) cascade, where StMAPKK1 (a MAPK kinase) may play a critical role in abiotic stress tolerance [5]. The MAPK cascade is a highly conserved signaling pathway in eukaryotes, consisting of three core components: MAPKKKs (MAP3Ks/MEKKs), MAPKKs (MKKs/MEKs), and MAPKs (MPKs) [6].

Plant MAPKKs are highly conserved dual-specificity kinases that phosphorylate MAPKs on Thr and Tyr residues in the TXY motif. The Arabidopsis genome contains 10 MAPKKs, which are classified into four groups. Unlike animals and yeast, plant MAPKKs have a unique activation loop motif (S/T-XXXXX-S/T) and a conserved MAPK-docking domain (K/R-K/R-K/R-X_1_-_6_-L-X-L/V/I) at their N-termini [7]. In plants, this cascade acts as a critical transduction module downstream of receptors and sensors, enabling cells to perceive and respond to both endogenous and external stimuli [8]. Upon activation, the pathway relays a signal through sequential phosphorylation events, amplifying the initial signal and triggering specific physiological and biochemical responses that regulate plant growth, development, and stress adaptation [9].

Genome sequencing has enabled the identification of MAPKK genes across diverse plant species. Arabidopsis (*Arabidopsis thaliana*) encodes 10 MAPKKs [7,10], while other species exhibit variations in gene number: rice (*Oryza sativa*) contains 8 MAPKKs [11], maize (*Zea mays*) has 9 MAPKKs [12], tomato (*Solanum lycopersicum*) and cucumber (*Cucumis sativus*) each possess 6 MAPKKs [13,14], wheat (*Triticum aestivum*) comprises 18 MAPKKs [15], banana (*Musa acuminate*) has been identified to have 10 MAPKKs [16], and in green Algae (*Chlamydomonas reinhardtii*) has only two MAPKKs (*MAPKK1* and *MAPKK2*) [17]. This comparative analysis underscores the conservation and divergence of MAPKK family members in plants. Furthermore, MAPKKs have been functionally characterized as key regulators of abiotic stress responses across diverse plant species. In *Arabidopsis*, *AtMEKK1*, *AtMKK2* are induced by cold and salt stress [8], while cucumber *CsMKK4* responds to heat treatment [14]. Similarly, strawberry (*Fragaria vesca*) shows upregulation of *FvMAPKK1/3/5/6/7* under high-temperature and salt stress conditions [18]. Similarly, in rice, the *OsMKK6-OsMPK3* signaling cascade regulates cold stress [19], and *OsMKK1/3/4/6* regulates high temperatures and other abiotic stresses [20]. In maize, *ZmKK4* mitigates salt stress under high NaCl accumulation [21], whereas in tomato, *SlMAPKK1/2/3/4/5* exhibit upregulation under heat, cold, salt, and drought stress conditions [13]. Moreover, *OsMKK6* enhances drought tolerance through stomatal regulation and reactive oxygen species (ROS) scavenging [18], *TaMKK2* maintains ion homeostasis under salinity [15], and *GhMKK5* activates antioxidant defenses in salt-stressed cotton [22]. Similarly, *CsMKK4* confers thermotolerance in cucumber [14], while *StMAPKK5* coordinately regulates heat, drought, and salt responses via antioxidant enhancement and photosynthetic maintenance in potato [23,24]. In addition, the MAPK cascade transduces nitrogen signals via RAF14/RAF79, which are ammonium-repressed and NIT2-independent, suggesting roles as algal nitrogen sensors. RAF14’s phosphorylation responds dynamically to N status, while its genomic clustering with RAF80/81 implies functional coordination. This pathway integrates nitrogen sensing with metabolic regulation and stress responses [17].

Comprehensive genome-wide analysis in potato has identified eight MAPKK genes, classified into distinct groups: Group A (*StMAPKK2* and *StMAPKK3*), Group B (*StMAPKK4, StMAPKK5, StMAPKK6*, and *StMAPKK8*), Group C (*StMAPKK7*), and Group D (*StMAPKK1*) [25]. However, functional characterization has thus far been limited, with only *StMAPKK5* from Group B being investigated and shown to play roles in potato responses to heat stress [23], drought, and salinity [24]. This study pioneers the functional analysis of *StMAPKK1*, a group D MAPKK in potato, revealing its unique thermotolerance role distinct from well-characterized group A/B MAPKKs in Solanaceae. Group D MAPKKs, characterized by distinct regulatory motifs (e.g., TDY phosphorylation sites), are less understood, despite their potential divergence in stress-specific functions. Unlike cold/drought-responsive MAPKKs (e.g., *AtMKK2*, *SlMKK2/4*, *NtMEK2*), *StMAPKK1* uniquely regulates heat stress adaptation. By bridging these gaps, this study provides a foundation for engineering climate-resilient potatoes through targeted manipulation of group D MAPKK pathways. Furthermore, our previous study demonstrated that *StMAPKK1* exhibits significant upregulation in potato tissues under heat stress (35 °C) across multiple time points by qRT-PCR analysis [23], providing a molecular basis for investigating its role in thermotolerance. Within this context, the present study analyzed the expression patterns of *StMAPKK1* in two potato cultivars, the heat-sensitive ‘*Atlantic*’ and the heat-tolerant ‘*Desiree*’, under different heat stress regimes (25 °C, 30 °C, and 35 °C). Furthermore, we systematically analyzed the specific thermotolerance functions mediated by *StMAPKK1*, including its effects on antioxidant activity, photosynthetic capacity, growth parameters, and the mRNA expression levels of key antioxidant enzyme genes and heat stress-responsive genes in potato plants.

## 2. Results

### 2.1. Sequence Alignment and Phylogenetic Analysis of StMAPKK1

**Figure 1:**
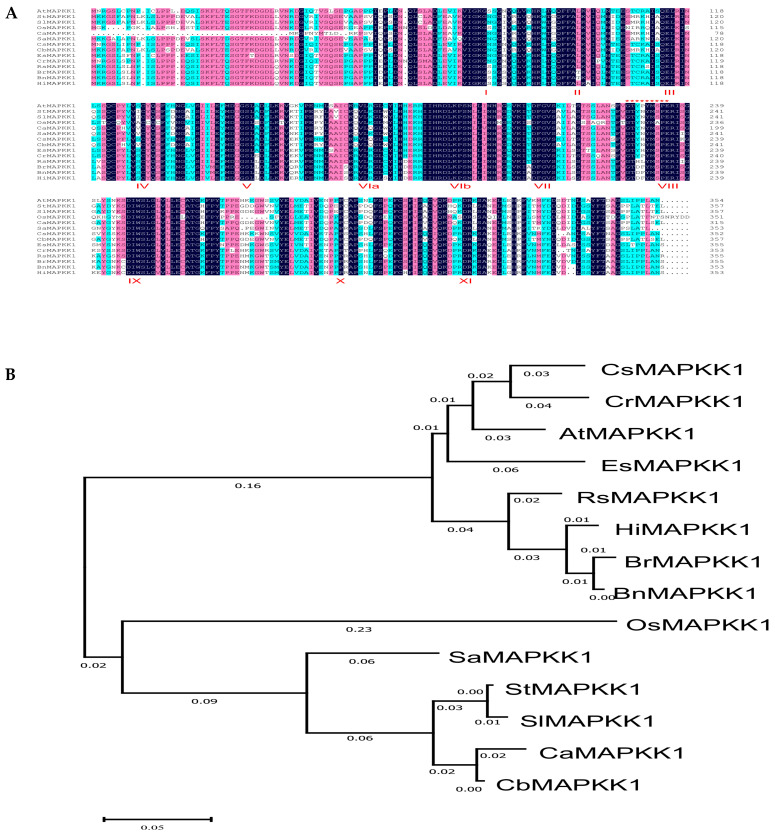
The protein sequence alignment and phylogenetic relationship of MAPKK1 proteins across different plant species. (**A**) The predicted amino acid residues alignment of Siberian oilseed (*Camelina sativa*) CsMAPKK1, pink shepherd’s-purse (*Capsella rubella*) CrMAPKK1, Arabidopsis (*Arabidopsis thaliana*) AtMAPKK1, saltwater cress (*Eutrema salsugineum*) EsMAPKK1, radish (*Raphanus sativus*) RsMAPKK1, Hoary Mustard (Hirschfeldia incana) HiMAPKK1, turnip mustard (*Brassica rapa*) BrMAPKK1, rapeseed (*Brassica napus*) BnMAPKK1, rice (Oryza sativa) OsMAPKK1, winged-seed sesame (*Sesamum alatum*) SaMAPKK1, potato (*Solanum tuberosum*) StMAPKK1, tomato (*Solanum lycopersicum*) SlMAPKK1, pepper (*Capsicum annuum*) CaMAPKK1, and Aji Amarillo (*Capsicum baccatum*) CbMAPKK1. The background colors indicate the degree of similarity among the amino acid sequences. The conserved subdomains are indicated at the bottom, using Roman numerals (I–XI). The first red line highlights the conserved consensus motif (GXGXXG), while the asterisks represent the unique catalytic domain (VGTxxYM(S/A) PEG). Different colors showed level of sequence alignment homology (light blue color; 50%, red color; 75%, and black color; 100% sequence alignment homology). (**B**) Neighbor-joining phylogenetic tree illustrating the relationships of MAPKK1 between potato and other plant species. The unrooted tree was constructed using the neighbor-joining method in MEGA 4.1. Bootstrap values exceeding 50% from 1000 replicates are shown at each branch.

The predicted amino acid residues alignment of Siberian oilseed (*Camelina sativa*) CsMAPKK1, pink shepherd’s-purse (*Capsella rubella*) CrMAPKK1, Arabidopsis (*Arabidopsis thaliana*) AtMAPKK1, saltwater cress (*Eutrema salsugineum*) EsMAPKK1, radish (*Raphanus sativus*) RsMAPKK1, Hoary Mustard (Hirschfeldia incana) HiMAPKK1, turnip mustard (*Brassica rapa*) BrMAPKK1, rapeseed (*Brassica napus*) BnMAPKK1, rice (Oryza sativa) OsMAPKK1, winged-seed sesame (*Sesamum alatum*) SaMAPKK1, potato (*Solanum tuberosum*) StMAPKK1, tomato (*Solanum lycopersicum*) SlMAPKK1, pepper (*Capsicum annuum*) CaMAPKK1, and Aji Amarillo (*Capsicum baccatum*) CbMAPKK1 is displayed in Figure 1A. The protein accession numbers of MAPKK1 from various plant species were identified using NCBI Protein BLAST (https://blast.ncbi.nlm.nih.gov/Blast.cgi?PAGE=Proteins, accessed on 10 December 2023), as listed in Table S1. All MAPKK1 proteins across plant species contained eleven conserved catalytic domains (I–XI) (Figure 1A). Comparative sequence analysis revealed that all examined MAPKK1 orthologs conserve two critical functional domains: the canonical GXGXXG phosphate-binding motif within Subdomain I, and the distinctive catalytic domain VGTxxYM(S/A) PEG in Subdomain VIII (Figure 1A). Phylogenetic analysis of plant MAPKK1 amino acid sequences revealed that StMAPKK1 clusters within a well-supported clade alongside SlMAPKK1, CsMAPKK1, CrMAPKK1, AtMAPKK1, EsMAPKK1, RsMAPKK1, HiMAPKK1, BrMAPKK1, BnMAPKK1, OsMAPKK1, SaMAPKK1, SlMAPKK1, CaMAPKK1, and CbMAPKK1, as displayed in Figure 1B, suggesting functional conservation across these orthologs. Furthermore, phylogenetic analysis revealed that StMAPKK1 shares high sequence similarity and clusters closely with SlMAPKK1 (*Solanum lycopersicum*), CaMAPKK1 (*Capsicum annuum*), and CbMAPKK1 (*Capsicum baccatum*), forming a well-supported Solanaceae-specific clade (Figure 1B). Interestingly, this group also showed significant affinity with SaMAPKK1 (*Sesamum alatum*) from the Pedaliaceae family. The strong phylogenetic conservation among these MAPKK1 homologs suggests they likely share functional similarities and may have undergone parallel evolutionary selection pressures within the clades.

### 2.2. Expression Profiling of StMAPKK1 Under Heat Stress in Potato Cultivars

**Figure 2:**
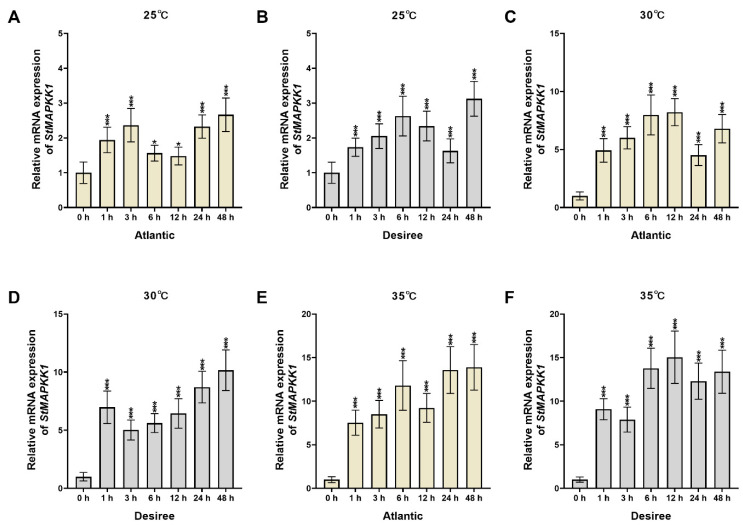
The expression patterns of *StMAPKK1* in the leaves of potato cultivars ‘*Atlantic*’ and ‘*Desiree*’ in response to induced heat (25 °C, 30 °C, and 35 °C), stress conditions at various time intervals (0, 1, 3, 6, 12, 24, and 48 h). (**A**,**B**), expression profile of *StMAPKK1* in ‘*Atlantic*’ and ‘*Desiree*’ cultivars at 25°C at various time points, (**C**,**D**), expression profile of *StMAPKK1* in ‘*Atlantic*’ and ‘*Desiree*’ cultivars at 30°C at various time points, and (**E**,**F**), expression profile of *StMAPKK1* in ‘*Atlantic*’ and ‘*Desiree*’ cultivars at 35°C at various time points. The data are presented as mean ± standard deviation. *p*-values (* *p* < 0.05, *** *p* < 0.001) were calculated through ordinary two-way ANOVA followed by Tukey’s multiple comparisons test with a sample size of n = 9.

We investigated the relative expression patterns of *StMAPKK1* in potato cultivars ‘*Atlantic*’ and ‘*Desiree*’ under heat stress conditions at 25 °C, 30 °C, and 35 °C across multiple time points (0, 1, 3, 6, 12, 24, and 48 h). Under 25 °C stress (Figure 2A,B), ‘*Atlantic*’ displayed a fluctuating upregulation pattern with increased expression at 1 and 3 h, followed by a decrease at 6 h, continued decline at 12 h, and significant enhancement at 24 and 48 h. In contrast, ‘*Desiree*’ showed an overall increasing trend characterized by initial upregulation (1, 3, and 6 h), subsequent downregulation (12 and 24 h), and peak expression at 48 h. At 30 °C (Figure 2C,D), ‘*Atlantic*’ maintained general upregulation despite fluctuations, with continuous increases from 1 to 12 h, a temporary decrease at 24 h, and recovery at 48 h. Similarly, ‘*Desiree*’ exhibited predominant upregulation with marked increase at 1 h, slight decrease at 3 h, followed by progressive elevation through 48 h, reaching maximum expression. Under 35 °C stress (Figure 2E,F), ‘*Atlantic*’ demonstrated strong upregulation with continuous increases from 1 to 6 h, a minor decrease at 12 h, and sustained high expression levels at 24 and 48 h (peaking at 48 h). ‘*Desiree*’ also showed significant upregulation: rapid increase at 1 h, slight decrease at 3 h, progressive rise to peak at 12 h, modest decline at 24 h, and subsequent increase at 48 h. These results demonstrate distinct temporal expression patterns of *StMAPKK1* between cultivars under heat stress, revealing genotype-specific thermoresponse characteristics. The differential regulation indicates *StMAPKK1*’s involvement in potato heat stress responses, with its expression patterns being modulated by cultivar-specific genetic backgrounds.

### 2.3. Subcellular Localization of StMAPKK1 and Generation of Transgenic Potato Lines

**Figure 3:**
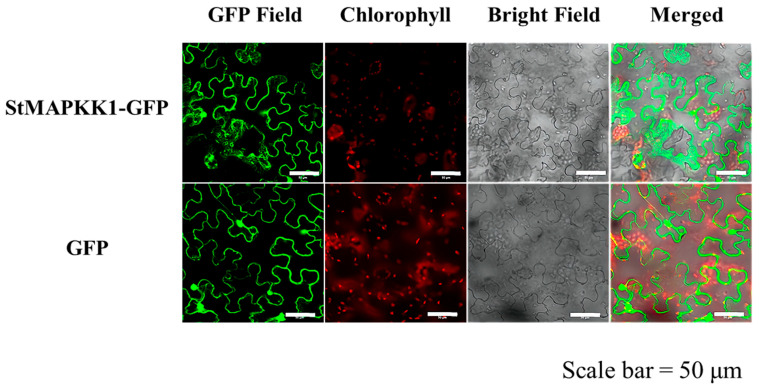
Subcellular localization of StMAPKK1-GFP fusion protein. Confocal laser scanning microscopy was conducted to analyze tobacco plants transformed with the *pCAM35*-GFP-*StMAPKK1* construct. The empty vector expressing only GFP was used as a control. Scale bar = 50 μm.

The subcellular localization of StMAPKK1 was analyzed via the *Agrobacterium tumefaciens* (strain GV3101)-mediated transient expression system in Nicotiana benthamiana leaf epidermal cells, and distinct distribution patterns were observed between the experimental and control groups. In cells transfected with the *pCAM35S*-GFP-*StMAPKK1* fusion construct (*StMAPKK1*-GFP group), GFP fluorescence (GFP Field) was predominantly localized to the plasma membrane and cytoplasmic compartments (Figure 3). In marked contrast, control cells expressing the empty vector *pCAM35S*-GFP (GFP group) exhibited GFP fluorescence widely distributed throughout the cytoplasm, nucleus, and plasma membrane (Figure 3). Chlorophyll autofluorescence (red) is shown to visualize chloroplasts. Bright field images and merged images representing the overlay of GFP fluorescence and chlorophyll autofluorescence signals are presented concurrently in Figure 3.

**Figure 4:**
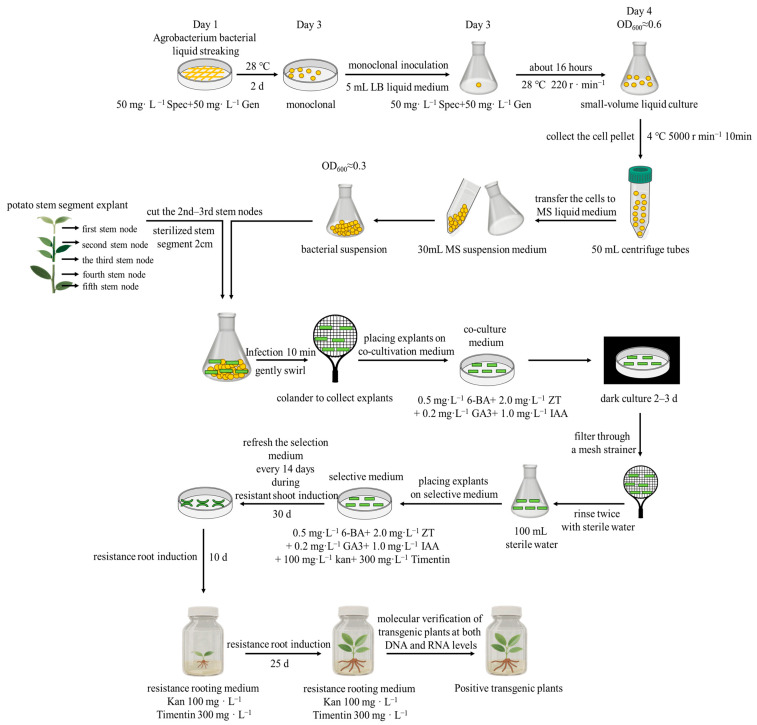
Schematic flowchart showing major steps involved in the generation of transgenic potato plants using Agrobacterium-mediated transformation methods.

To investigate the role of *StMAPKK1* in heat stress responses, we generated stable transgenic potato lines with either *StMAPKK*1-OE or RNAi-knockdown in two cultivars (heat-sensitive ‘*Atlantic*’ and heat-tolerant ‘*Desiree*’). The Agrobacterium-mediated genetic transformation methodology is illustrated in Figure 4. The transformation efficiencies were 20.0% for ‘*Atlantic’* (7 transgenic events from 35 explants) and 25.7% for ‘*Desiree’* (9 events from 35 explants), with 28 and 26 explants discarded, respectively. Then, the success of genetic transformation was confirmed by qRT-PCR analysis: OE lines exhibited significant upregulation of *StMAPKK1* expression (*** *p* < 0.001). For subsequent experiments, we selected three independent high-expression lines from each cultivar (‘*Atlantic*’: OE-1/3/5; ‘*Desiree*’: OE-1/2/4; Figure S1A,B) and three effective RNAi-knockdown lines (‘*Atlantic*’: RNAi-1/2/5; ‘*Desiree*’: RNAi-2/4/6; Figure S1C,D), which showed significant suppression of *StMAPKK1* expression. This rigorous selection of transgenic lines with markedly different expression levels (*StMAPKK1*-OE and RNAi-knockdown lines) provides a solid foundation for precisely dissecting the regulatory functions of *StMAPKK1* in heat stress responses.

### 2.4. StMAPKK1-Mediated Signaling Enhances Thermotolerance and Biomass Accumulation

**Figure 5:**
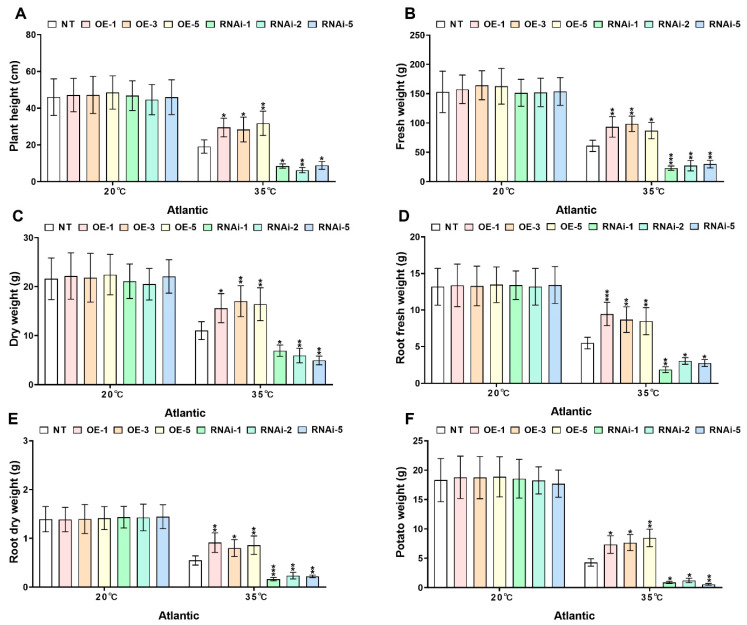
*StMAPKK1* modulates potato growth parameters of cultivar ‘*Atlantic*’; (**A**) plant height, (**B**) fresh weight, (**C**) dry weight, (**D**) root fresh weight, (**E**) root dry weight, and (**F**) potato weight, after exposure to 20 °C and 35 °C of heat stress treatments. NT, non-transgenic plants; OE, *pBI121*-EGFP-*StMAPKK1*-transgenic plants (OE-1, OE-3, and OE-5); RNAi, *pART*-*StMAPKK1*- RNAi-transgenic plants (RNAi-1, RNAi-2 and RNAi-5). The data are presented as mean ± standard deviation. *p*-values (* *p* < 0.05, ** *p* < 0.01, *** *p* < 0.001) were calculated through ordinary two-way ANOVA followed by Tukey’s multiple comparisons test with a sample size of n = 9.

**Figure 6:**
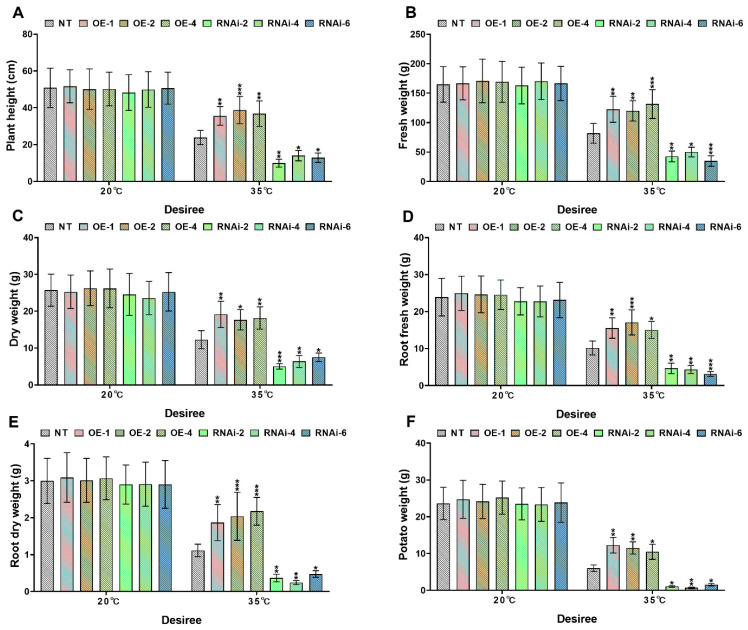
*StMAPKK1* modulates potato growth parameters of cultivar ‘*Desiree*’; (**A**) plant height, (**B**) fresh weight, (**C**) dry weight, (**D**) root fresh weight, (**E**) root dry weight, and (**F**) potato weight, after exposure to 20 °C and 35 °C of heat stress treatments. NT, non-transgenic plants; OE, *pBI121*-EGFP-*StMAPKK1*-transgenic plants (OE-1, OE-2, and OE-4); RNAi, *pART*-*StMAPKK1*- RNAi-transgenic plants (RNAi-2, RNAi-4 and RNAi-6). The data are presented as mean ± standard deviation. *p*-values (* *p* < 0.05, ** *p* < 0.01, *** *p* < 0.001) were calculated through ordinary two-way ANOVA followed by Tukey’s multiple comparisons test with a sample size of n = 9.

Under controlled conditions (20 °C), no significant differences (*p* > 0.05) were observed in growth parameters-including plant height (Figure 5 and Figure 6A), fresh weight (Figure 5 and Figure 6B), dry weight (Figure 5 and Figure 6C), root fresh weight (Figure 5 and Figure 6D), root dry weight (Figure 5 and Figure 6E), and tuber weight per plant (Figure 5 and Figure 6F)—between transgenic potato lines and NT lines of both ‘*Atlantic’* and ‘*Desiree*’ cultivars. However, heat stress (35 °C) significantly inhibited growth across all genotypes, with *StMAPKK1*-OE lines demonstrating superior thermotolerance. In the ‘*Atlantic*’ cultivar, compared to NT controls, the OE lines (OE-1, OE-3 and OE-5) maintained significantly higher plant height (Figure 5A), fresh weight (Figure 5B), dry weight (Figure 5C), root fresh weight (Figure 5D), root dry weight (Figure 5E), and tuber yield per plant (Figure 5F) under heat stress. In contrast, the RNAi-knockdown lines (RNAi-1, RNAi-2 and RNAi-5) exhibited increased heat sensitivity, showing reduced plant height and significantly decreased values in other growth-related parameters (fresh weight, dry weight, root fresh weight, root dry weight, and tuber weight per plant) (* *p* < 0.05, ** *p* < 0.01, *** *p* < 0.001) (Figure 5A–F). A similar trend was observed in the ‘*Desiree*’ cultivar under heat stress (35 °C). The *StMAPKK1-*OE lines (OE-1, OE-2 and OE-4) displayed superior performance in plant height (Figure 6A), fresh weight (Figure 6B), dry weight (Figure 6C), root fresh weight (Figure 6D), root dry weight (Figure 6E), and tuber yield per plant (Figure 6F) compared to NT controls. Conversely, the RNAi-knockdown lines (RNAi-2, RNAi-4, and RNAi-6) showed significantly lower values in all growth parameters than NT plants (* *p* < 0.05, ** *p* < 0.01, *** *p* < 0.001) (Figure 6A–F). These results demonstrate that *StMAPKK1* enhances thermotolerance in potato plants, effectively alleviating the negative impacts of heat stress on growth and yield.

### 2.5. StMAPKK1 Enhances Potato Thermotolerance by Modulating Antioxidant Defense, Osmoprotectant Biosynthesis, Oxidative Stress Markers, and Chlorophyll Stability

**Figure 7:**
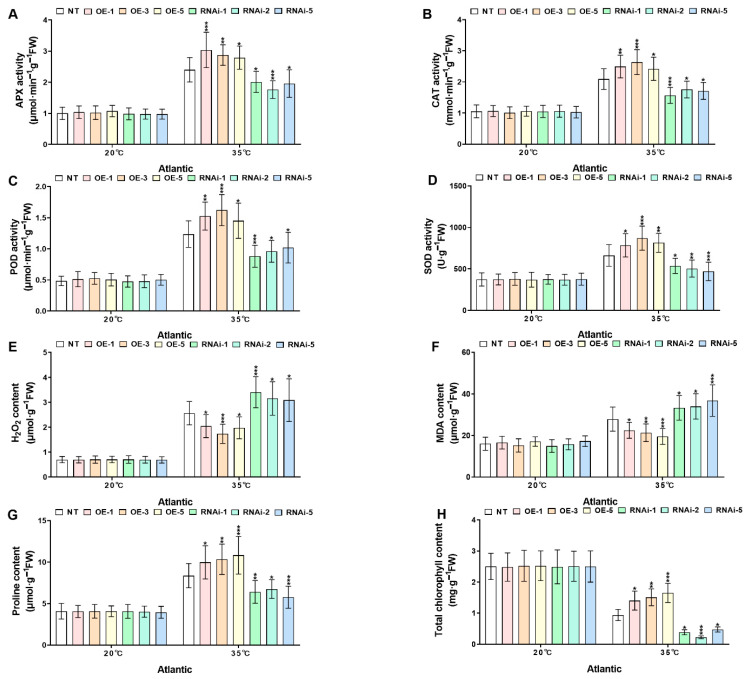
*StMAPKK1* modulates potato physiological indices of cultivar ‘*Atlantic*’; (**A**) APX activity, (**B**) CAT activity, (**C**) POD activity, (**D**) SOD activity, (**E**) H_2_O_2_ content, (**F**) MDA content, (**G**) Proline content, and (**H**) Total chlorophyll content, after exposure to 20 °C and 35 °C of heat stress treatments. NT, non-transgenic plants; OE, *pBI121*-EGFP-*StMAPKK1*-transgenic plants (OE-1, OE-3, and OE-5); RNAi, *pART*-*StMAPKK1*-RNAi-transgenic plants (RNAi-1, RNAi-2 and RNAi-5). The data are presented as mean ± standard deviation. *p*-values (* *p* < 0.05, ** *p* < 0.01, *** *p* < 0.001) were calculated through ordinary two-way ANOVA followed by Tukey’s multiple comparisons test with a sample size of n = 9.

**Figure 8:**
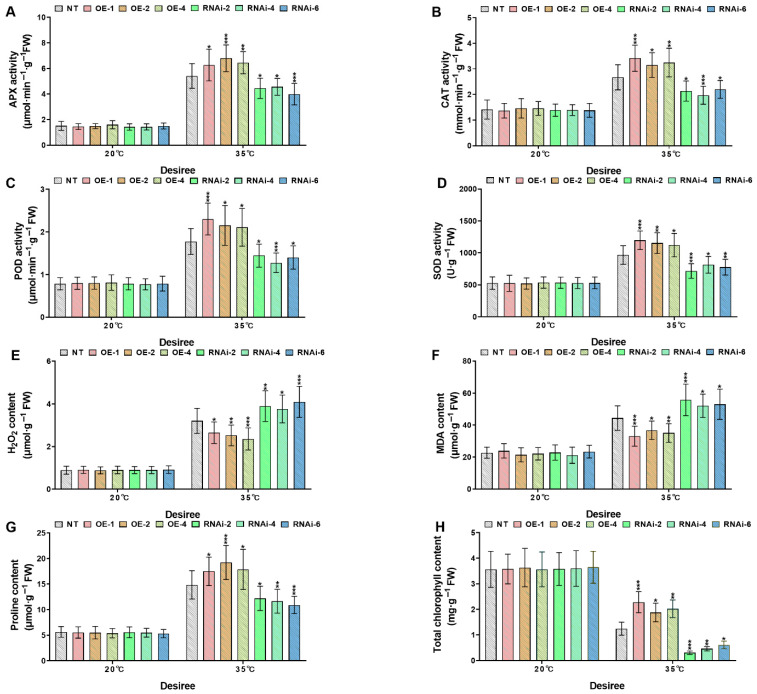
*StMAPKK1* modulates potato physiological indices of cultivar ‘*Desiree*’; (**A**) APX activity, (**B**) CAT activity, (**C**) POD activity, (**D**) SOD activity, (**E**) H_2_O_2_ content, (**F**) MDA content, (**G**) Proline content, and (**H**) Total chlorophyll content, after exposure to 20 °C and 35 °C of heat stress treatments. NT, non-transgenic plants; OE, *pBI121*-EGFP-*StMAPKK1*-transgenic plants (OE-1, OE-2, and OE-4); RNAi, *pART*-*StMAPKK1*-RNAi-transgenic plants (RNAi-2, RNAi-4 and RNAi-6). The data are presented as mean ± standard deviation. *p*-values (* *p* < 0.05, ** *p* < 0.01, *** *p* < 0.001) were calculated through ordinary two-way ANOVA followed by Tukey’s multiple comparisons test with a sample size of n = 9.

To elucidate the role of *StMAPKK1* in heat stress adaptation, transgenic (*StMAPKK1*-OE lines and RNAi-knockdown lines) and NT potato lines of cultivars ‘*Atlantic*’ and ‘*Desiree*’ were subjected to heat stress conditions (20 °C and 35 °C). Physiological and biochemical analyses assessed antioxidant enzymes (APX, CAT, POD, SOD), oxidative stress markers (H_2_O_2_, MDA), osmoregulatory proline, and chlorophyll content as key indicators of thermotolerance. Under control conditions (20 °C), no significant differences (*p* > 0.05) were observed between transgenic and NT potato plants of both cultivars. However, when heat stress treatment was increased to 35 °C, compared to NT plants, *StMAPKK1*- OE lines in both cultivars of potato exhibited enhanced and robust upregulation (* *p* < 0.05, ** *p* < 0.01, *** *p* < 0.001) of APX (Figure 7 and Figure 8A), CAT (Figure 7 and Figure 8B), POD (Figure 7 and Figure 8C), and SOD (Figure 7 and Figure 8D) proline (Figure 7 and Figure 8E), and chlorophyll levels (Figure 7 and Figure 8H) due to *StMAPKK1-* mediated stress signaling, leading to preventive antioxidant and proline synthesis to mitigate oxidative damage, and an increased chlorophyll content protect against photo-oxidative damage. Unlike the antioxidant enzyme systems, proline and chlorophyll content, the oxidative stress markers, such as H_2_O_2_ (Figure 7 and Figure 8F) and MDA (Figure 7 and Figure 8G) accumulation, were diminished in *StMAPKK1-*OE lines, compared to NT lines, which inhibits oxidative signaling and lipid peroxidation. Conversely, RNAi-knockdown lines showed reduced antioxidant enzyme activities, proline and chlorophyll levels, while increased H_2_O_2_ and MDA contents were detected relative to NT lines. The compromised thermotolerance in RNAi-knockdown lines demonstrates that *StMAPKK1*-mediated signaling is essential for maintaining antioxidant defense, osmoprotection, and chloroplast stability under heat stress by coordinately suppressing ROS accumulation and oxidative damage.

### 2.6. StMAPKK1 Regulates the Stress-Responsive Gene Network to Confer Heat Tolerance in Potato

**Figure 9:**
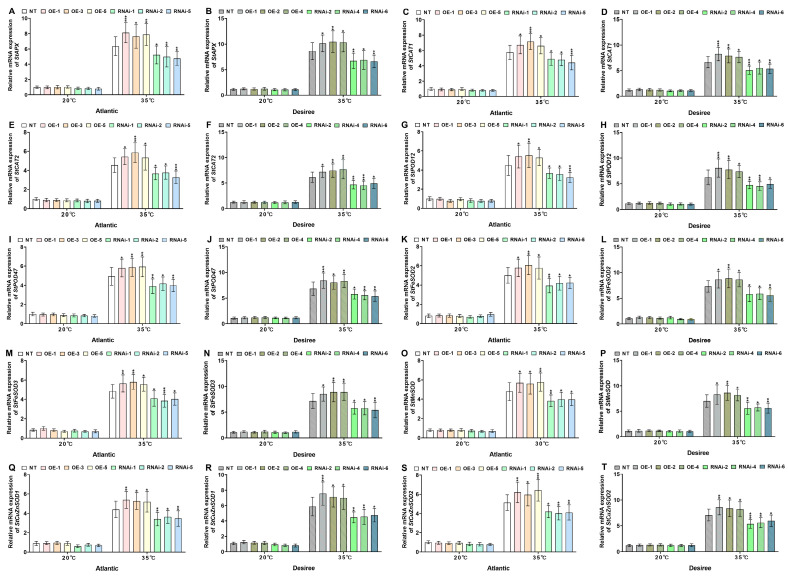
*StMAPKK1* regulates the relative mRNA expression of potato plants of cultivar ‘*Atlantic*’ ‘*Desiree*’; (**A**,**B**) *StAPX*, (**C**,**D**) *StCAT1*, (**E**,**F**) *StCAT2*, (**G**,**H**) *StPOD12*, (**I**,**J**) *StPOD47*, (**K**,**L**) *StFeSOD2*, (**M**,**N**) *StFeSOD3*, (**O**,**P**) *StMnSOD*, (**Q**,**R**) *StCuZnSOD1*, and (**S**,**T**) *StCuZnSOD2*, respectively, after exposure to 20 °C and 35 °C of heat stress. In the ‘*Atlantic*’ cultivar: NT, non-transgenic plants; OE, *pBI121*-EGFP-*StMAPKK1*-transgenic plants (OE-1, OE-3, and OE-5); RNAi, *pART*-*StMAPKK1*-RNAi-transgenic plants (RNAi-1, RNAi-2, and RNAi-5). In the ‘*Desiree*’ cultivar: NT, non-transgenic plants; OE, *pBI121*-EGFP-*StMAPKK1*-transgenic plants (OE-1, OE-2, and OE-4); RNAi, *pART*-*StMAPKK1*-RNAi-transgenic plants (RNAi-2, RNAi-4, and RNAi-6). The data are presented as mean ± standard deviation. *p*-values (* *p* < 0.05, ** *p* < 0.01, *** *p* < 0.001) were calculated through ordinary two-way ANOVA followed by Tukey’s multiple comparisons test with a sample size of n = 9.

Through systematic analysis of key antioxidant gene expression patterns (*StAPX, StCAT1*, *StCAT2*, *StPOD12*, *StPOD47*, *StFeSOD2*, *StFeSOD3*, *StMnSOD*, *StCuZnSOD1,* and *StCuZnSOD2*) in *StMAPKK1*-OE, RNAi-knockdown, and NT potato lines (cultivars ‘*Atlantic*’ and ‘*Desiree*’) under heat stress conditions (35 °C), this study elucidates the role of *StMAPKK1* in transcriptional regulation of antioxidant genes during thermal stress adaptation. Under optimal conditions (20 °C), no significant differential expression of antioxidant genes was observed among all tested genotypes (Figure 9A–T). However, heat stress (35 °C) induction triggered pronounced transcriptional responses with *StMAPKK1*-OE lines exhibiting coordinated upregulation of peroxidase-related genes, such as *StAPX* (Figure 9A,B), *StPOD12* (Figure 9G,H), and *StPOD47* (Figure 9I,J). Similarly, an upregulation was also observed in catalase-related genes, including *StCAT1* (Figure 9C,D) and *StCAT2* (Figure 9E,F) in *StMAPKK1*-OE plants of *‘Atlantic*’ and ‘*Desiree*’ potato cultivars. Additionally, genes related to superoxide dismutase isoforms, comprised *StFeSOD2* (Figure 9G,H), *StFeSOD3* (Figure 9I,J), *StMnSOD* (Figure 9K,L), *StCuZnSOD1* (Figure 9M,N), and *StCuZnSOD2* (Figure 9O,P), exhibited an increased expression level in *StMAPKK1*-OE lines of both cultivars, compared to NT lines of potatoes. On the contrary, RNAi lines of both ‘*Atlantic*’ and ‘*Desiree*’ cultivars exhibited significant downregulation in expression of all aforementioned antioxidant enzyme-associated genes, owing to impaired signaling cascade upon *StMAPKK1* knockdown. This conclusively demonstrates *StMAPKK1*’s role as a regulatory hub in the ROS-scavenging network. This trend intensified under severe stress (35 °C), where OE lines maintained robust upregulation of all assessed genes, while RNAi lines displayed further suppression. Furthermore, under control conditions (20 °C), no differential expression of heat stress-responsive genes (*StHSFA3*, *StHSP20*, *StHSP70*, *StHSP90*) was observed in either cultivar ( ‘*Atlantic*’ and ‘*Desiree*’), as shown in Figure S2A–H. However, at 35 °C, *StMAPKK1*-OE plants exhibited significant upregulation of these heat stress-responsive genes, including *StHSFA3* (Figure S2A,B), *StHSP20* (Figure S2C,D), *StHSP70* (Figure S2E,F), and *StHSP90* (Figure S2G,H), whereas RNAi-knockdown lines showed downregulation, compared to NT control, suggesting that *StMAPKK1* positively regulates heat shock protein-mediated thermotolerance in potato.

### 2.7. StMAPKK1 Maintains Photosynthesis Under Heat Stress in Potato

**Figure 10:**
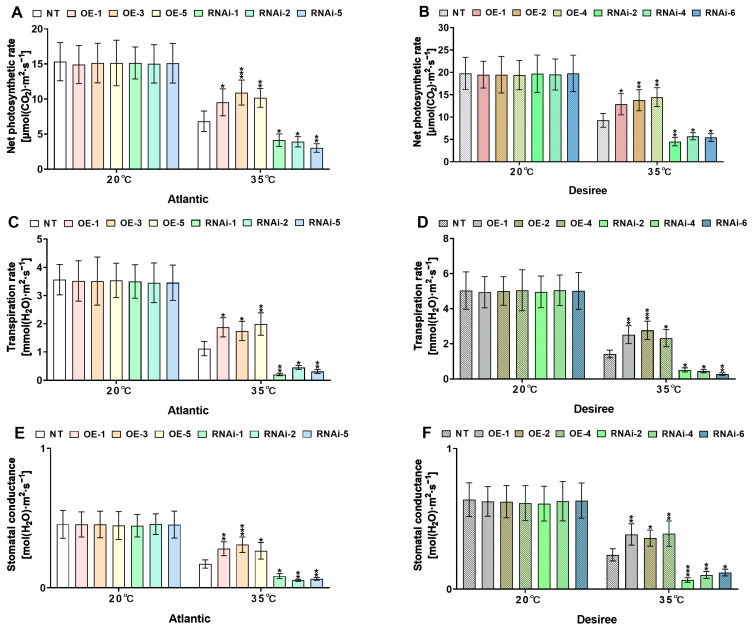
*StMAPKK1* regulates the photosynthetic traits of potato plants of cultivars, ‘*Atlantic*’ and ‘*Desiree*’; (**A**,**B**) net photosynthetic rates, (**C**,**D**) transpiration rates, and (**E**,**F**) stomatal conductance, respectively, after exposure to 20 °C and 35 °C of heat stress treatments. In the ‘*Atlantic*’ cultivar: NT, non-transgenic plants; OE, *pBI121*-EGFP-*StMAPKK1*-transgenic plants (OE-1, OE-3, and OE-5); RNAi, *pART*-*StMAPKK1*-RNAi-transgenic plants (RNAi-1, RNAi-2, and RNAi-5). In the ‘*Desiree*’ cultivar: NT, non-transgenic plants; OE, *pBI121*-EGFP-*StMAPKK1*- transgenic plants (OE-1, OE-2, and OE-4); RNAi, *pART*-*StMAPKK1*-RNAi-transgenic plants (RNAi-2, RNAi-4, and RNAi-6). The data are presented as mean ± standard deviation. *p*-values (* *p* < 0.05, ** *p* < 0.01, *** *p* < 0.001) were calculated through ordinary two-way ANOVA followed by Tukey’s multiple comparisons test with a sample size of n = 9.

We systematically evaluated the photosynthetic parameters of transgenic (*StMAPKK1*-OE and RNAi-knockdown) and non-transgenic lines of potato cultivars ‘*Atlantic*’ and ‘*Desiree*’ under normal temperature (20 °C) and heat stress (35 °C) conditions, revealing genotype-specific differences in their responses to heat stress (35 °C). Under control conditions (20 °C), no significant differences (*p* > 0.05) were observed in photosynthetic indices, including Pn, E, and Gs, between the transgenic (*StMAPKK1*-OE and RNAi-knockdown) lines and NT counterparts of either the heat-sensitive cultivar ‘*Atlantic*’ or the heat-tolerant cultivar ‘*Desiree*’ (Figure 10A–F). When subjected to heat stress treatment (35 °C), all experimental plants of both ‘*Atlantic*’ and ‘*Desiree*’ exhibited declines in the aforementioned photosynthetic traits. However, compared to the NT controls, the *StMAPKK1*-OE lines maintained significantly higher levels of Pn (Figure 10A,B), E (Figure 10C,D), and Gs (Figure 10E,F). Conversely, the RNAi-knockdown lines, with suppressed *StMAPKK1* expression, showed significant reductions in photosynthetic activity (Pn, Gs, and E). These findings demonstrate the regulatory role of the *StMAPKK1* gene in potato thermotolerance, which confers heat resistance to plants by maintaining multiple gas exchange parameters under severe heat stress (35 °C).

## 3. Discussion

The role of the MAPKK gene family in responding to abiotic stress has been extensively studied across numerous plants and crops [10,12,15,17,20,21,25,26]. Under global climate change, abiotic stresses like heat significantly threaten potato production by impairing physiological processes and reducing yield quality [26,27,28,29]. The MAPKK gene family plays a crucial role in model plants and other important crops [13,14,20,30,31,32] under heat stress through MAPK signaling, yet its function in potato thermotolerance remains limited to *StMAPKK5,* a B group MAPKK gene [23]. Here, we investigate the role of *StMAPKK1*, a D group MAPKK gene, in heat stress adaptation to address this knowledge gap. Under control conditions (20 °C), neither the *StMAPKK1*-OE lines nor the RNAi knockdown lines showed adverse phenotypic effects. Moreover, their physiological and biochemical indices as well as developmental processes were not significantly different from those of non-transgenic (NT) plants. This indicates that the regulation of *StMAPKK1* has no obvious pleiotropic effects on the basic growth of potatoes.

Multiple sequence alignment of StMAPKK1 with 13 orthologs revealed conserved plant MAPKK features: the GXGXXG nucleotide-binding motif (subdomain I) and catalytic VGTxxYM(S/A) PEG domain (subdomain VIII), demonstrating evolutionary conservation across all eleven kinase subdomains (I-XI) as depicted in Figure 1A. These features ensure signaling specificity, while motif variations may influence substrate recognition, necessitating further study. Our sequence alignment analysis, supported by previous research, confirms that StMAPKKs contain two critical conserved motifs: (1) the catalytic domain VGTxxYM(S/A) PEG, characteristic of MAPKK kinases, and (2) the phosphorylation site motif S/T-x5-S/T [24], which is universally conserved across plant species, including wheat, *Arabidopsis*, and rice [15]. The presence of these evolutionarily preserved domains in *StMAPKK1* strongly demonstrates the functional conservation of MAPKKs in plant signaling pathways. Furthermore, phylogenetic analysis classifies potato MAPKKs into four groups (A-D). Groups C (StMAPKK7) and D (StMAPKK1) each contain one member, while group A includes StMAPKK2/3. Group B is the largest (StMAPKK4/5/6/8), representing half of all potato MAPKKs, suggesting significant functional diversification in stress-related pathways [25]. Our phylogenetic analysis revealed that StMAPKK1 clusters most closely with its ortholog in tomato, which suggests it may share the highest degree of functional homology with tomato SlMAPKK1 among all species in the cluster (Figure 1B). Our qRT-PCR analysis revealed that *StMAPKK1* exhibits an upregulated and cultivar-specific expression pattern in ‘*Atlantic*’ and ‘*Desiree*’ cultivars of potato leaves under various heat (25 °C, 30 °C, and 35 °C) stress treatments (Figure 2A–F), suggesting its potential role in thermotolerance mechanisms. *CsMKK3* and *CsMKK6* exhibited rapid induction, showing significant upregulation at 1, 2, and 8 h, suggesting their direct involvement in early heat stress signaling. *CsMKK4* displayed biphasic regulation, with initial downregulation (1–4 h) followed by late upregulation (8 h), implying a potential role in secondary stress responses. *CsMKK2-1, CsMKK2-2*, and *CsMKK9* were consistently downregulated, indicating they may function as negative regulators of heat stress responses [14]. In potatoes, although *StMAPKK1* also showed differential expression profiles at different time points (1–48 h), it was up-regulated under high-temperature stress, especially at 35 °C, with a significant up-regulation. Similarly, studies in tomato further validate our findings, demonstrating significant upregulation of multiple MAPKK genes under. heat stress. Notably, *SlMAPKK42* and *SlMAPKK60* exhibited remarkable induction (>100-fold) across all examined tissues, including roots, stems, leaves, flowers, and fruits [13]. These findings provide strong support that *StMAPKK1* confers heat tolerance in potato plants, demonstrating that MAPKK family members function as pivotal regulators in plant thermotolerance responses. The MAPKK family plays a crucial role in the MAPK signaling cascade, which regulates diverse cellular processes, including stress responses, growth, and development. Furthermore, the MAPKK (MKK/MEK) family is divided into four major groups (A, B, C, D) based on sequence homology and functional divergence [33].

Mitogen-activated protein kinase (MAPK) is activated in the cytoplasm by extracellular signals and subsequently translocates to the nucleus. Its direct activator, MAPKK, remains cytoplasmic to relay signals from the plasma membrane to MAPK [34]. Previous studies on MAPKK protein localization have demonstrated their predominant distribution in the plasma membrane, cytoplasm, and nucleus. For instance, *MtMAPKK4* in *Medicago truncatula* was shown to localize to the membrane, cytoplasm, and nucleus via green fluorescent protein (GFP) fusion assays [35] Consistent with PSORT predictions, a previous experimental validation confirmed that StMAPKK5-EGFP localizes to the nucleus, cytoplasm, and cytoplasmic membrane [24]. Intriguingly, this pattern differs from StMAPKK1, which we demonstrated to localize specifically to the cytoplasm and plasma membrane by GFP signals of the *pCAM35S*-GFP-*StMAPKK1* construct (Figure 3). The distinct localization of StMAPKK1 suggests its primary role in membrane-associated signal transduction, while StMAPKK5 may have additional nuclear regulatory functions. This divergence highlights functional specialization within the MAPKK family, with StMAPKK1 likely playing a key role in transmitting extracellular signals from the plasma membrane. Further studies should explore how these spatially segregated kinases coordinate in stress response or developmental pathways.

Heat stress reduces plant growth and yield by impairing photosynthesis, cell division, and reproduction. It decreases plant height, biomass, and root-shoot ratios while damaging leaves and shortening grain filling [36]. The cotton *GhMAPKK3* has been established as a critical regulator of drought tolerance, functioning through its control of root architecture modification. This kinase mediates water conservation through rapid stomatal closure while simultaneously enhancing root system development for improved hydraulic conductivity, collectively maintaining plant productivity under water deficit conditions [37]. Building upon these findings in cotton, our investigation of the potato *StMAPKK1* revealed parallel mechanisms in thermotolerance regulation. Transgenic *StMAPKK1*-OE lines demonstrated superior growth performance and yield stability under elevated temperatures (35 °C), whereas RNAi-knockdown lines exhibited marked heat sensitivity with significant reductions in biomass accumulation and tuber production (Figure 5 and Figure 6). The conserved nature of MAPKK family members in coordinating stress adaptation through growth trait regulation is once again evidenced by this cross-species (cotton–potato) functional consistency under divergent stress conditions. Furthermore, the functional conservation of MAPKK-mediated stress responses extends beyond thermo- and drought tolerance, as evidenced by research in maize chilling adaptation. Cai and his colleagues (2014) demonstrated that *ZmKK1* overexpression confers chilling tolerance through enhanced seedling vigor and root elongation, mediated by downstream MAPK cascade activation that upregulates both stress-responsive gene networks and antioxidant defense systems [38]. This comparison (cotton–potato–maize) reveals a remarkable model where MAPKK isoforms across species coordinate stress resilience through regulating growth and biomass. *StMAPKK1* overexpression in ‘*Atlantic*’ and ‘*Desiree*’ potato cultivars enhanced heat stress (35 °C) tolerance by significantly increasing antioxidant activity, osmoprotectant accumulation, and chlorophyll content (Figure 7 and Figure 8). Additionally, *StMAPKK1*-overexpressing lines exhibited reduced oxidative stress markers, preserving membrane integrity and minimizing lipid peroxidation under heat stress. In contrast, *StMAPKK1* knockdown lines showed the opposite effects across all measured parameters compared to NT controls under identical stress conditions. Our results showed consistency with a previous study that investigated under drought stress, which demonstrated that *PtMKK4*-overexpressing plants displayed a significant reduction in hydrogen H_2_O_2_ accumulation alongside elevated activity of key antioxidant enzymes, including superoxide SOD, CAT, and POD, when compared to wild-type controls [39]. Overexpression of *GhMKK3* in cotton conferred enhanced drought tolerance, as demonstrated by significantly reduced accumulation of MDA and H_2_O_2_ under drought stress. In contrast, vector control (Vec.) plants exhibited severe oxidative damage, with higher MDA and H_2_O_2_ levels compared to *GhMKK3*-overexpressing lines [37]. Moreover, *ZmMKK1* transgenic tobacco improved cold tolerance in rice by enhancing antioxidant enzymes (APX, SOD, POD, and CAT), proline and chlorophyll accumulation, and reducing the oxidative stress markers (H_2_O_2_, MDA) in transgenic plants, while the opposite is true for Vec. plants for all the above-mentioned physiological parameters [38]. Collectively, these findings demonstrate that *StMAPKK1*, *ZmMKK1*, *GhMKK3*, and *PtMKK4* function as key regulators of abiotic stress tolerance by attenuating oxidative damage through the modulation of antioxidant defense systems. Specifically, these MAPKK family members enhance the activity of ROS-scavenging enzymes and osmoprotectant ability while reducing the accumulation of cytotoxic oxidative markers. This conserved functional role across diverse plant species (*Solanum tuberosum*, *Zea mays*, *Gossypium hirsutum*, and *Populus trichocarpa*) provides persuasive evidence for the pivotal involvement of the MAPKK gene family in plant stress adaptation. Furthermore, we examined the expression profiles of key stress-responsive marker genes, including *StAPX*, *StCAT1/2*, *StPOD12/47*, *StFeSOD2/3*, *StMnSOD*, and *StCuZnSOD1/2* (Figure 9), as well as *StHSFA3*, *StHSP20*, *StHSP70*, and *StHSP90* (Figure S2) under both control and heat stress conditions in ‘*Atlantic*’ and ‘*Desiree*’ potato cultivars. Hasan M K et al. [40] observed that under high-temperature stress, tomato plants in elevated CO_2_ ( 800μmol mol^-1^) environment showed upregulation of heat shock protein gene expression in both wild-type WT and transgenic lines overexpressing *COMT1* (*COMT1*-OE). In this study, we also found that *StHSP20/70/90* genes also showed an upregulated expression response pattern in NT and *StMAPKK1*-OE potato lines under heat stress.

Comparative analysis revealed a significant upregulation of these genes in *StMAPKK1*-OE plants, whereas RNAi-mediated suppression of *StMAPKK1* resulted in their pronounced downregulation relative to NT control plants in both cultivars. Our study aligned with a prior investigation, which reported that the overexpression of *ZmMKK3* accelerated stress-responsive gene induction, with *NtAPX* upregulation occurring by 12 h and sustained through 48 h relative to WT plants. Likewise, *NtSOD* expression was constitutively higher in OE lines than in WT under osmotic stress [41]. Moreover, under normal conditions, transgenic plants exhibited slightly higher expression of POD and CAT compared to controls. However, under salt and drought stress, these genes were significantly upregulated in transgenic plants, demonstrating that *ZmMKK1* enhances stress tolerance by modulating the expression of genes related to antioxidant enzymes (POD, CAT) [38]. These results indicate that *StMAPKK1* (heat stress), *ZmMKK1* (osmotic stress), and *ZmMKK3* (drought and salt stress) enhance abiotic stress tolerance through elevated antioxidant activity and stress gene expression. A key regulatory mechanism in plant stress response is the heat shock factors-heat shock proteins (HSF–HSP) signaling pathway, where plant transcription factor HSFs (HSFA2, HSFA3, HSF4A, HSF5B) activate the expression of HSPs (HSP20, HSP21, HSP70, HSP80, HSP90) to protect cells from thermal and other abiotic stresses [42]. The upregulation of *StHSFA3*, *StHSP20*, *StHSP70*, and *StHSP90* in *StMAPKK1*-overexpressing plants aligns (Figure S2) with established reports of *MAPKK5*-mediated activation of HSF/HSP transcription [23]. We postulate that *StMAPKK1* and *StMAPKK5* may share common downstream regulatory components coordinating thermotolerance responses in potato, a premise warranting further experimental validation.

**Figure 11:**
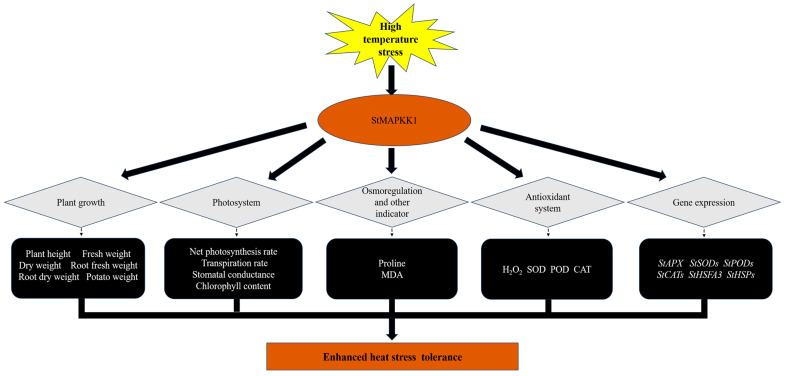
A proposed model illustrating the role of *StMAPKK1* in potato plants’ responses to heat stress. Under high-temperature stress, *StMAPKK1* enhances tolerance by regulating growth, biomass, photosystem efficiency, oxidative markers, and antioxidant enzymes while promoting osmoregulatory compounds to reduce ROS accumulation. It also mediates the upregulation of stress-responsive genes, contributing to improved stress adaptation.

Heat stress impairs photosynthesis by damaging PSII, reducing Rubisco activity, and increasing photorespiration. While initially boosting transpiration, prolonged heat causes stomatal closure, lowering conductance and CO_2_ uptake. This dual effect, reduced carbon fixation and restricted gas exchange, hinders plant growth [43]. Our findings demonstrate that under elevated temperature stress (35 °C), *StMAPKK1-*OE potato plants exhibited significantly enhanced Pn, E, and Gs compared to NT controls of both ‘*Atlantic*’ and ‘*Desiree*’ cultivars. Conversely, *StMAPKK1*-knockdown lines displayed a marked reduction in these photosynthetic parameters relative to NT plants (Figure 10). *GhMKK1* overexpression conferred enhanced drought tolerance without affecting stomatal aperture under normal conditions. During drought, stomata of wild-type plants nearly closed completely, while transgenic plants maintained significantly wider apertures. After recovery, transgenic stomata were wider than wild-type plants [44]. Similarly, under cold stress conditions, the detached leaves of *ZmMKK1*-overexpressing plants exhibited significantly lower transpiration water loss compared to Vec. plants following exposure to a 12 °C treatment [38]. These results suggest that *StMAPKK1*, *GhMKK1*, and *ZmMKK1* play a critical regulatory role in maintaining photosynthetic efficiency and stomatal function under abiotic stress, potentially through modulation of stomatal behavior and photochemical processes. The *StMAPKK1*-OE enhances heat tolerance in potato plants through multiple protective mechanisms: regulating plant growth and biomass, protecting photosynthesis, controlling oxidative stress (via antioxidant enzymes like APX, SOD, POD, CAT), promoting osmoprotectants (e.g., proline), and activating stress-responsive genes (Figure 11). By coordinating these responses, *StMAPKK1* helps plants withstand heat stress while reducing cellular damage.

The current study has several limitations and future perspectives that need to be addressed to advance our understanding of *StMAPKK1*-mediated thermotolerance in potatoes. First, while the functional validation of *StMAPKK1* transgenic lines was performed under controlled conditions, field trials under natural high-temperature stress are essential to fully evaluate its agronomic potential. Second, although *StMAPKK1* contributes to thermotolerance, its precise position within the MAPK signaling cascade remains unclear. Future research should focus on identifying the upstream and downstream kinases of *StMAPKK1*, along with its interacting regulatory partners, to elucidate its regulatory network under heat stress. Although, previous yeast two-hybrid screening identified five proteins interacting with StMAPKK1 in potato: (1) an O-glycosyl hydrolase, (2) a RING-H2 RHE protein, (3) cyanate esterase, (4) an ARF GTPase-activating factor, and (5) a C2 domain-containing protein [45]. The specific mechanism related to protein–protein interaction under abiotic stress will be explored in our subsequent investigation. Third, the potential cross-talk between StMAPKK1 and other stress-responsive pathways, such as ABA, calcium signaling, nitrogen metabolism, and other abiotic stressors (salt, drought, cold, heavy metal, etc.) requires systematic investigation. Integrated multi-omics approaches, including transcriptomics, proteomics, and phosphoproteomics, could help uncover these interactions. Addressing these questions will deepen our understanding of the comprehensive role *StMAPKK1* plays in mediating both heat stress-associated MAPK cascades and other regulatory networks.

## 4. Materials and Methods

### 4.1. Plant Materials and Stress Treatments

The experimental potato materials, consisting of the heat-sensitive cultivar ‘*Atlantic*’ and heat-tolerant cultivar ‘*Desiree*’, were provided by Gansu Agricultural University and maintained at the South Subtropical Crops Research Institute of the Chinese Academy of Tropical Agricultural Sciences. The experimental protocol involved transferring uniform shoot tips from tissue-cultured seedlings of both cultivars to MS medium for 4 weeks under controlled conditions (16 h light/8 h dark photoperiod, 2800 lux illumination, 20 °C). Subsequently, seedlings were transplanted into sterilized substrate soil and acclimatized for 2 weeks in growth chambers, maintaining identical photoperiod and temperature conditions at 70–75% relative humidity. Uniform plants were then transplanted into 26 cm × 27 cm × 18 cm pots containing a 1:1 (*v*/*v*) mixture of nutrient soil and vermiculite, and cultivated in Zhanjiang (21°11′43″ N, 110°34′56″ E) for 35 days with soil moisture maintained at 70–75%. Weekly irrigation was performed using 100 mL of pH 5.8 nutrient solution containing 0.20 mmol/L FeSO_4_, 2.57 mmol/L KH_2_PO_4_, 2.08 mmol/L MgSO_4_, 1.29 mmol/L (NH_4_)_2_SO_4_, and 9.89 mmol/L KNO_3_.

To investigate *StMAPKK1* responses to heat stress, plants were subjected to temperature treatments of 25 °C, 30 °C, and 35 °C (with 20 °C as a control). Leaf samples were collected at 0, 1, 3, 6, 12, 24, and 48 h post-treatment for mRNA expression analysis. The experimental design included 504 potted plants, representing two cultivars, four temperature treatments (20 °C, 25 °C, 30 °C, 35 °C), seven time points, and triplicate biological and technical replicates per treatment. According to the above growth conditions, uniformly grown transgenic (*StMAPKK1*-OE and RNAi-knockdown) and non-transgenic (NT) plants of both potato cultivars were subjected to high-temperature stress (35 °C), while the control group remained at 20 °C, with other conditions unchanged. Physiological and photosynthetic parameters were measured 48 h after treatment, with 3 biological and 3 technical replicates per group. To detect the growth indices of transgenic (*StMAPKK1*-overexpressing and RNAi-knockdown) and NT lines of both cultivars under 35 °C heat stress, healthy and uniform plants were transplanted into a soil-vermiculite mixture (1:1, *v*/*v*) and cultivated for 5 weeks with soil moisture maintained at 70–75%, as previously described. Then, uniform transgenic and non-transgenic lines were selected to grow for 6 weeks under 20 °C and 35 °C heat stress, respectively, with 3 biological and 3 technical replicates per group.

### 4.2. Phylogenetic Analysis and Sequence Comparison

Evolutionary relationships were reconstructed in MEGA X (version 4.1) using the Neighbor-Joining algorithm, with branch support evaluated through 1000 bootstrap replicates [46]. The multiple-sequence alignment of the conserved subdomains was performed using DNAMAN software (version 10, Lynnon Biosoft, San Ramon, CA, USA).

### 4.3. Generation of StMAPKK1 Transgenic Potato Plants

Following the established protocol [47], the *StMAPKK1* coding sequence was cloned into *pBI121*-EGFP, generating the overexpression construct. To achieve gene knockdown, an RNAi expression vector was prepared according to the method described by [48]. All primer sequences are listed in Table S2. The transformation procedure involved culturing recombinant *Agrobacterium tumefaciens* strain LBA4404 in antibiotic-supplemented LB medium (50 mg/L each of gentamicin and spectinomycin) at 28 °C for 48 h. Bacterial cells were then concentrated by centrifugation at 5000 rpm for 10 min and adjusted to OD600 = 0.3 in MS liquid medium. Sterilized stem segments (2 cm length) were immersed in this suspension for 10 min before transfer to co-culture medium containing plant growth regulators (0.5 mg/L 6-BA, 2.0 mg/L ZT, 0.2 mg/L GA3, and 1.0 mg/L IAA) and maintained in darkness for 2–3 days. Selection of transformed tissues was performed on media supplemented with 100 mg/L kanamycin and 300 mg/L Timentin, with sub-culturing every 14 days. Developing shoots were subsequently transferred to rooting medium (MS + 7.4 g/L agar + 30 g/L sucrose + 300 mg/L Timentin + 100 mg/L kan, pH = 5.8) for complete plant regeneration.

### 4.4. qRT-PCR Analysis of Gene Expression

Total RNA was isolated from plant samples using the TRIzol reagent (Invitrogen, Carlsbad, CA, USA). cDNA synthesis was performed with the First-Strand cDNA Synthesis Kit (TransGen Biotech, Beijing, China) according to the manufacturer’s instructions. qPCR analysis was carried out on a LightCycler 480 II instrument (Roche, Basel, Switzerland) using SYBR Green chemistry (Takara, Shiga, Japan). Each 20 μL reaction contained 100 ng of template cDNA, 0.5 μM of gene-specific primers (Table S2), and 10 μL of 2× SYBR Premix Ex Taq. The thermal cycling conditions consisted of initial denaturation at 94 °C for 3 min, amplification consisted of 36 cycles of 94 °C for 45 s, 59 °C for 34 s, and 72 °C for 1 min. For normalization of qRT-PCR data, the gene Stef1α served as an internal reference. Expression levels of target genes were calculated using the comparative CT method (2^−ΔΔCt^) as described by Livak and Schmittgen [49]. The analysis included three independent biological replicates, with each sample measured in triplicate (technical replicates) to ensure data reliability. All primer sequences used for amplification are detailed in Supplementary Table S2.

### 4.5. Subcellular Localization of StMAPKK1

The full-length coding sequence of *StMAPKK1* was cloned into the *pCAM35*-GFP vector (primers listed in Table S2). Subsequently, the resulting recombinant plasmid *pCAM35*-GFP-*StMAPKK1* and the empty vector control *pCAM35*-GFP were independently introduced into *Agrobacterium tumefaciens* strain GV3101. Transient expression in tobacco (*Nicotiana benthamiana*) epidermal cells was performed according to established protocols [50]. Fluorescence detection parameters were optimized for distinct cellular components: chlorophyll autofluorescence was monitored at 640 nm excitation/675 nm emission, while GFP-tagged StMAPKK1 signals were detected at 488 nm excitation/510 nm emission. All images were captured and processed using Olympus Fluoview imaging software.

### 4.6. Analysis of Growth Parameters

Under 35 °C heat stress conditions, growth parameters of transgenic lines (*StMAPKK1*-OE and RNAi-knockdown lines) and NT controls from two potato cultivars were analyzed, including plant height, tuber yield per plant, plant and root fresh weights, and plant and root dry weights. Plant height was defined as the vertical distance from the soil surface to the apical meristem of the main stem in pot-grown plants. Tuber yield per plant was quantified as the total fresh weight of tubers produced by individual plants per pot after 6 weeks of cultivation under the specified conditions, while plant and root fresh weights were measured immediately upon harvest. Dry weights were determined following a modified protocol described previously [51], where freshly harvested plant and root samples from transgenic and NT lines were placed in a constant-temperature drying oven, subjected to heat deactivation at 105 °C for 10 min, dried at 80 °C until constant weight, and subsequently weighed after cooling in a desiccator.

### 4.7. Measurement of Photosynthetic Parameters and Chlorophyll Content

The 35-day-old potted transgenic plants (*StMAPKK1*-OE lines and RNAi-knockdown lines) along with NT controls were subjected to 35 °C heat stress for 48 h (with other growth conditions unchanged and 20 °C as the control), after which photosynthetic parameters were immediately measured. Using an LI-6400XT portable photosynthesis system (Li-COR, Lincoln, NE, USA), we determined Pn, E, and Gs. All measurements were conducted between 9:30 and 11:30 AM on the fourth fully expanded leaf under controlled chamber conditions with the following parameters: CO_2_ concentration (400 μmol/mol), photosynthetic photon flux density (1500 μmol·m^−2^·s^−1^), and relative humidity (70–75%). Chlorophyll content was measured according to the protocol described earlier [52], with detailed experimental procedures provided in Supplementary Data S1.

### 4.8. Biochemical Analysis of Stress Markers and Antioxidant Enzyme Activities

The 35-day-old potted transgenic plants (*StMAPKK1*-OE lines and RNAi-knockdown lines) and NT plants were subjected to 35 °C heat stress for 48 h (with other growth conditions unchanged and 20 °C as the control), and samples were immediately collected for analysis of the following biochemical parameters: proline [53], MDA [54], H_2_O_2_ contents [55], and antioxidant enzyme activities including APX [56], CAT [57], SOD [58], POD [59]. All assays were performed using the previous established protocol [60], with detailed experimental procedures provided in Supplementary Data S1.

### 4.9. Statistical Analysis

Statistical analyses were conducted with GraphPad Prism Version 9 (GraphPad Software, San Diego, CA, USA) and IBM SPSS Statistics 19.0 (IBM Corporation, Chicago, IL, USA). Quantitative data were expressed as means ± standard deviation (SD). Graphical representations of results, including histograms and line graphs, were generated through GraphPad Prism. Appropriate statistical tests were selected based on experimental design: one-way ANOVA followed by Tukey’s post hoc test or Dunnett’s T3 procedure for group comparisons, and two-way ANOVA with Sidak’s correction for multiple comparisons.

## 5. Conclusions

This study elucidates the function of a novel Group D MAPKK gene, *StMAPKK1*, in potato thermotolerance. Our findings demonstrate that *StMAPKK1* is responsive to diverse heat stresses, and its overexpression enhances antioxidant enzyme activity, reduces oxidative damage, promotes proline and chlorophyll accumulation, and upregulates the expression of antioxidant genes and heat stress-responsive genes, consequently improving photosynthetic capacity and biomass accumulation under 35 °C heat stress. These results significantly expand our understanding of the physiological and biological functions of the *StMAPKK1* gene in the plant heat stress response.

## Data Availability

The datasets used and/or analyzed during the current study are available from the corresponding author upon reasonable request.

## Supplementary Materials

The following supporting information can be downloaded at https://www.mdpi.com/article/10.3390/plants14152289/s1, Figure S1. The relative mRNA expression levels of *StMAPKK1* in two potato cultivars: (A and B) *StMAPKK1-*OE lines; (C and D) RNAi-knockdown lines. Figure S2. *StMAPKK1*-mediated regulation of heat-responsive genes in potato cultivars ‘*Atlantic*’ and ‘*Desiree*’. Table S1. Protein IDs of MAPKK1 in different plant species. Table S2. Sequences of primers used in the present study. Supplementary Data S1. Assessment of various physiological indicators in response to heat stress in two cultivars of potato.

## Abbreviations

The following abbreviations are used in this manuscript:

MAPKK	Mitogen-activated protein kinase kinase
OE	Overexpressing
RNAi	RNA interference
NT	Non-transgenic
qRT-PCR	Quantitative real-time polymerase chain reaction
APX	Ascorbate peroxidase
CAT	Catalase
SOD	Superoxide dismutase
POD	Peroxidase
MDA	Malondialdehyde
H_2_O_2_	Hydrogen peroxide
HSFA3	Heat shock transcription factor A3
HSP	Heat shock protein
ROS	Reactive oxygen species
Vec.	Vector control
