# Supplementary material for: StMAPKK1 Enhances Thermotolerance in Potato (Solanum tuberosum L.) by Enhancing Antioxidant Defense and Photosynthetic Efficiency Under Heat Stress
Source: Plants (Basel). 2025 Jul 24;14(15):2289. doi: 10.3390/plants14152289 (PMC12348817; doi:10.3390/plants14152289)
Supplement: Supplementary file 1 [file plants-14-02289-s001.zip › plants-3758349-supplementary.pdf]

Title: *StMAPKK1* Enhances Thermotolerance in Potato (*Solanum tuberosum* L.) by Enhancing Antioxidant Defense and Photosynthetic Efficiency under Heat Stress

Supplementary Materials

1. Supplementary Figures

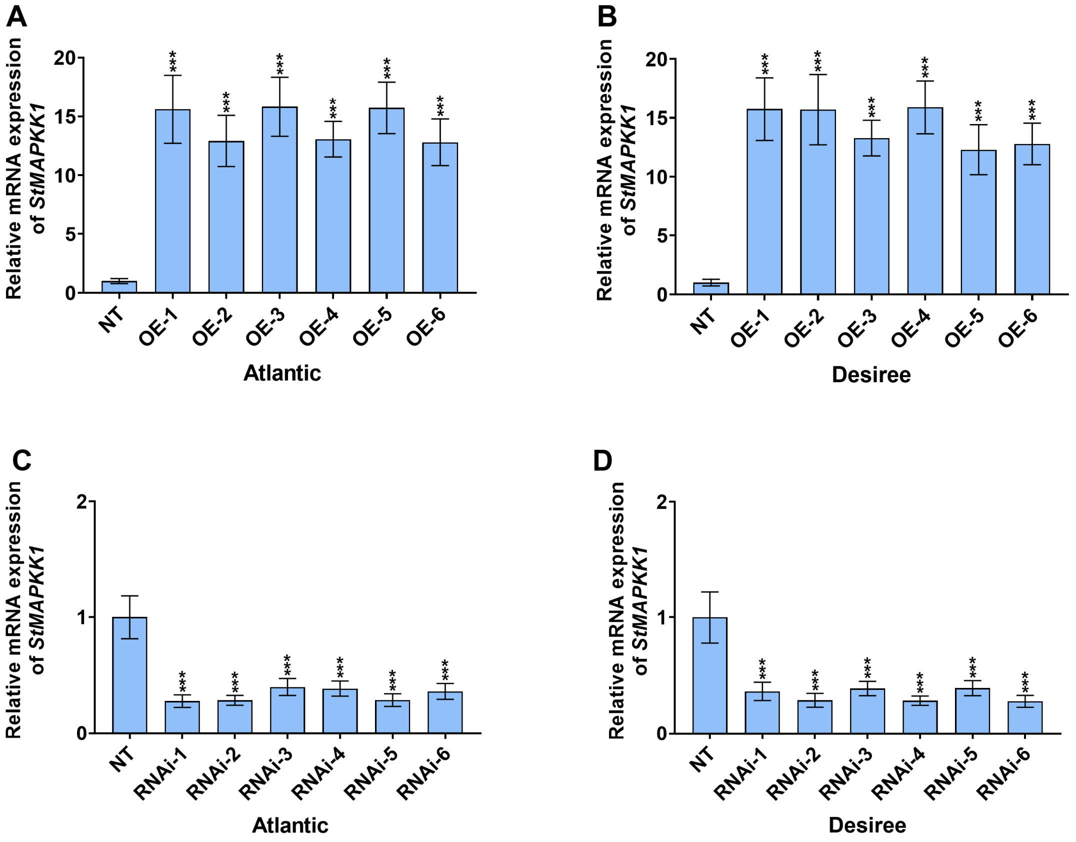

Figure S1. The relative mRNA expression levels of *StMAPKK1* in two potato cultivars: (A and B) *StMAPKK1*-OE lines; (C and D) RNAi-knockdown lines. In the '*Atlantc*' cultivar: NT, non-transgenic plants; OE, *pBI121*-EGFP-*StMAPKK1*-transgenic plants (OE-1, OE-2, OE-3, OE-4, OE-5, and OE-6); RNAi, *pART-StMAPKK1*-RNAi-transgenic plants (RNAi-1, RNAi-2, RNAi-3, RNAi-4, RNAi-5, and RNAi-6). In the '*Desiree*' cultivar: NT, non-transgenic plants; OE, *pBI121*-EGFP-*StMAPKK1*-transgenic plants (OE-1, OE-2, OE-3, OE-4, OE-5, and OE-6); RNAi, *pART-StMAPKK1*-RNAi-transgenic plants (RNAi-1, RNAi-2, RNAi-3, RNAi-4, RNAi-5, and RNAi-6). The data are presented as mean ± standard deviation. P-values (***$P < 0.001$) were calculated through ordinary two-way ANOVA followed by Tukey's multiple comparisons test with a sample size of n = 9.

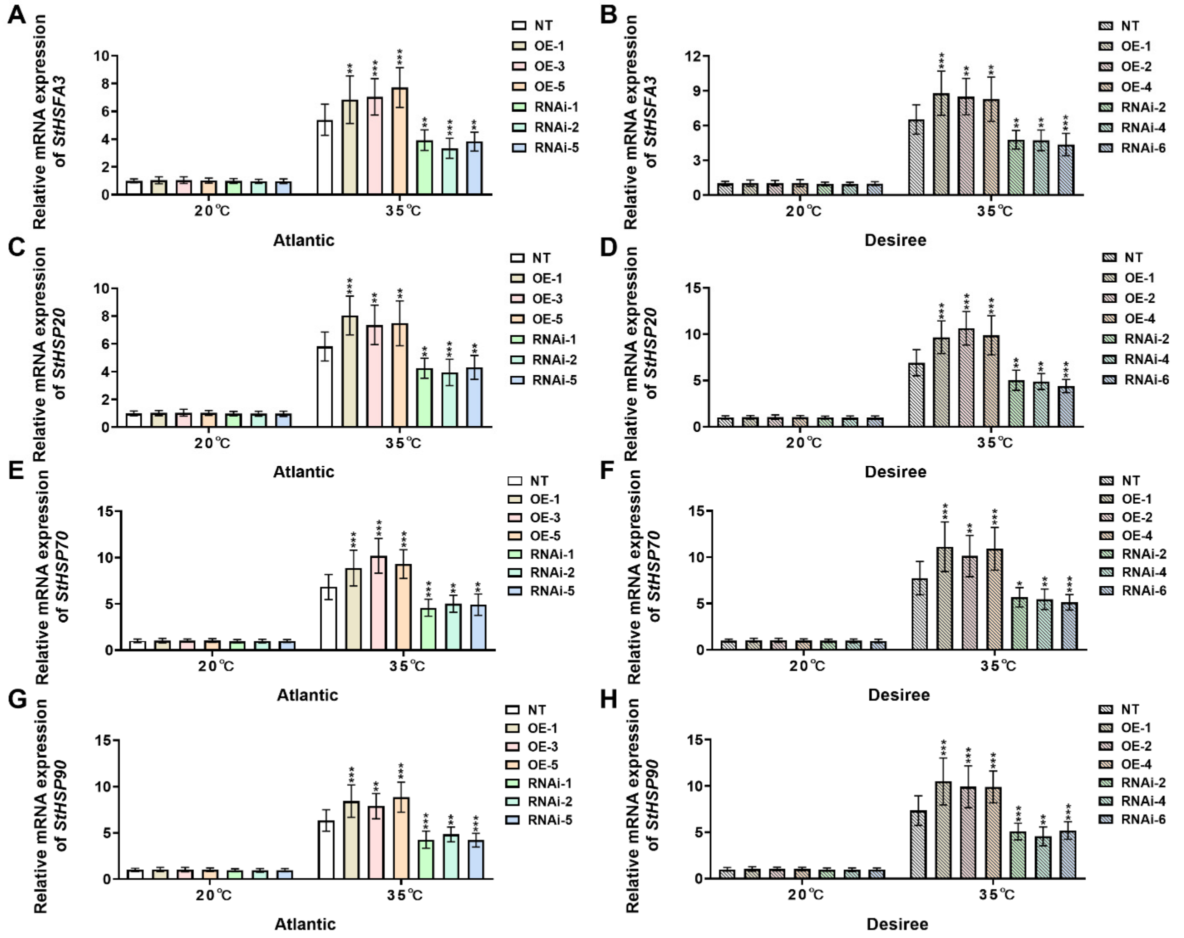

Figure S2. *StMAPKK1*-mediated regulation of heat-responsive genes in potato cultivars '*Atlantic*' and '*Desiree*'; (**A, B**) *StHSFA3*; (**C, D**) *StHSP20*; (**E, F**) *StHSP70*; (**G, H**) *StHSP90* expression under 20°C (control) vs. 35°C (heat stress). In the '*Atlantic*' cultivar: NT: non-transgenic plants; OE, *pBI121*-EGFP-*StMAPKK1*-transgenic plants (OE-1, OE-3, and OE-5); RNAi, *pART*-*StMAPKK1*-RNAi-transgenic plants (RNAi-1, RNAi-2, and RNAi-5). In the '*Desiree*' cultivar: NT: non-transgenic plants; OE, *pBI121*-EGFP-*StMAPKK1*-transgenic plants (OE-1, OE-2, and OE-4); RNAi, *pART*-*StMAPKK1*-RNAi-transgenic plants (RNAi-2, RNAi-4, and RNAi-6). The data are presented as mean ± standard deviation. P-values (*$*P < 0.05$, $**P < 0.01$, $***P < 0.001$*) were calculated through ordinary two-way ANOVA followed by Tukey's multiple comparisons test with a sample size of n = 9.

2. Supplementary Tables

Table S1. Protein IDs of the MAPKK1 in different plant species.

**Plant species**	**Protein**	**Protein ID**
Arabidopsis thaliana	AtMAPKK1	NP_194337.1
Solanum tuberosum	StMAPKK1	XP_006353547.1
Solanum lycopersicum	SlMAPKK1	NP_001304158.1
Capsicum annuum	CaMAPKK1	PHT60936.1
Capsella rubella	CrMAPKK1	XP_023633964.1
Raphanus sativus	RsMAPKK1	XP_018482656.1

Brassica rapa	BrMAPKK1	XP_009137576.1
Brassica napus	BnMAPKK1	XP_013706405.1
Hirschfeldia incana	HiMAPKK1	KAJ0263257.1
Oryza sativa	OsMAPKK1	XP_015644525.1
Sesamum alatum	SaMAPKK1	KAK4432886.1
Camelina sativa	CsMAPKK1	XP_010438800.1
Capsicum baccatum	CbMAPKK1	PHT52164.1
Eutrema salsugineum	EsMAPKK1	XP_006413236.2

The above-mentioned proteins can be directly accessed through the National Center for Biotechnology Information (NCBI database) (https://www.ncbi.nlm.nih.gov/guide/).

Table S2. Sequences of primers used in the present study.

**Gene ID**	**Gene**	**Forward (5'-3')**	**Reverse (5'-3')**
**Primers for overexpression:**			
XM_006353485.2	*StMAPKK1*	CTCGACATGAAGAAAGGATCTTTT GCT	GTCGACTAGCTCAGTAAGTGTTGC CAAT
**Primers for RNA interference expression:**			
XM_006353485.2	*StMAPKK1*	AGGATCTTTTGCTCCTAATCTT	ATCTGAATAACCTTGAGAGCG
**Primers for subcellular localization:**			
XM_006353485.2	*StMAPKK1*	ATGAAGAAAGGATCTTTTGCT	TAGCTCAGTAAGTGTTGCCAAT
**Primers for qRT-PCR:**			
XM_006347752.2	*StEf1α*	GGTTGTATCTCTTCCGATAAAGGC	GGTTGTATCTCTTCCGATAAAGGC
AB041343	*StAPX*	CTCCTCTGTGATCCTGCTTTC	GAGAGTGTCAAGTGAGCCTTAG
AY442179	*StCAT1*	GCACAGGGATGAGGAGATCG	CTTCTCACGTTTGCCACTGC
XM_006340770.2	*StCAT2*	GCACAGGGATGAGGAGATCG	CTTCTCACGTTTGCCACTGC
XM_006358985.2	*StPOD12*	CGGCCTTCTTCGTCTTCACT	AAACGACTCTACCGCAGTCC
XM_006350750.2	*StPOD47*	AGTCTGAGCAGGCCTTTGAC	GCCCATTTTACGCATGGCTT
XM_006358116.2	*StFeSOD2*	GCAGCCAAATTCAGCACACT	GGACCAGCTTTCCTCGCTAA
XM_006350307.2	*StFeSOD3*	TGCTGCCCAGGTATGGAATC	CCTCTCTGCTCAAGACGAGC
XM_006358693.2	*StMnSOD*	TAGACGTTTGGGAACACGCA	CTCTTCAGGGGCACTCGTTT
XM_049521383.1	*StCuZnSOD1*	CCTCCAACAGGTCACTGCTC	TCAGGTCACCCTTGAATGGC
AF354748	*StCuZnSOD2*	TGTGGCACCATCCTCTTCAC	TCCTGTTGACATGCAGCCAT
XM_006341106.1	*StHSFA3*	CAGCTTTGTTCGACAGCTTAATAC	CAAATGCCTCTTCCCTCTCAA
JX576239	*StHSP20*	GGAGAGAGGAATGTGGAGAAAG	CGCATTCTCCGGAAGTCTAAA

Z11982.1	*StHSP70*	GTGTTGGTGTATGGCAAAACGA	AGCAACTTGATTCTTGGCTGC
HS106768.1	*StHSP90*	CAGTGGTATCAACGCAGAGTAA	TCCTTCACAGACTTGTCATTCTT
XM_006353485.2	*StMAPKK1*	GAAGTTGCAGCTCCTTCAGT	GCGAAAAATTGCCCTGTCCA

The above-mentioned genes can be directly accessed through the National Center for Biotechnology Information (NCBI database) (https://www.ncbi.nlm.nih.gov/guide/).

3. Supplementary data 1

3.1. Assessment of various physiological indicators in response to heat stress in two cultivars of potato.

The following physiological indicators were measured as listed below.

3.1.1. Antioxidant enzyme activity determination

For enzyme extraction, 200 mg of frozen leaf tissue was mechanically homogenized in 4 mL of chilled 50 mM potassium phosphate buffer (pH 7.0). The extraction buffer contained 2 mM sodium EDTA and 1% (w/v) polyvinylpyrrolidone (PVPP) to enhance protein stability and remove phenolic compounds. Following homogenization, the samples were centrifuged at 10,000 × g for 10 minutes at 4°C to pellet cellular debris. The clarified supernatants were carefully transferred to fresh micro-centrifuge tubes and immediately stored at -80°C to preserve enzyme activity until analysis. To ensure experimental reproducibility, all enzymatic assays were performed using independently prepared extracts from three separate biological replicates. Enzyme activity measurements were conducted within two weeks of sample preparation to minimize degradation effects.

3.1.2. APX activity

The enzymatic activity of APX was evaluated by monitoring the oxidation rate of ascorbic acid at 290 nm wavelength, following the methodology established by Nakano and Asada (1981). The assay system (1 mL total volume) contained the following components, including 50 mM potassium phosphate buffer (pH 7.0), 1 mM EDTA-Na_2 (as a stabilizing agent), 0.5 mM freshly prepared ascorbic acid (substrate), 0.1 mM hydrogen peroxide (H_2O_2, reaction initiator), and 25 µL of enzyme extract. The reaction was started by adding H_2O_2, and the decrease in absorbance was recorded for 2 minutes at 25°C. APX activity was calculated using the molar extinction coefficient of 2.8 mM^{-1} cm^{-1} for oxidized ascorbate. One unit of APX activity was defined as the amount of enzyme required to oxidize 1 µmol of ascorbate per minute under the specified assay conditions.

3.1.3. POD activity

POD activity was determined spectrophotometrically by measuring the oxidation of guaiacol at 470 nm, following the established protocol (Maehly and Chance, 1954). The 3 mL reaction system contained 2.84 mL of 10 mM potassium phosphate buffer (pH 7.0), 50 µL of 20 mM guaiacol solution, and 90 µL of enzyme extract. The enzymatic reaction was initiated by adding 20 µL of 40 mM H_2O_2. The increase in absorbance was monitored for 2 minutes at 25°C. POD activity was measured using the guaiacol extinction coefficient of 26.6 mM^{-1} cm^{-1}, with one enzyme unit defined as the amount required to produce 1 µmol of tetra-guaiacol per minute under the specified conditions.

3.1.4. SOD activity

SOD activity was determined by measuring the inhibition of nitroblue tetrazolium (NBT) reduction in a photochemical reaction, adapted from Giannopolitis and Ries (1977). The 3 mL reaction mixture contained 50 mM potassium phosphate buffer (pH 7.8), 0.1 mM EDTA (pH 8.0), 14.9 mM methionine, 63 μM NBT, 90 μL of enzyme extract, and 8 μM riboflavin. The reaction was initiated by exposing the mixture to 4,000 lux light for 5 min, followed by immediate transfer to darkness to stop the reaction. Two control reactions were included: one kept in complete darkness and another exposed to light. The absorbance was recorded at 560 nm using a spectrophotometer. One unit (U) of SOD activity was defined as the enzyme quantity causing 50% inhibition of NBT reduction under the assay conditions.

3.1.5. CAT activity

CAT enzyme activity was measured spectrophotometrically by monitoring the decrease in absorbance at 240 nm resulting from H_2O_2 decomposition, according to the method described by Aebi (1984). The assay mixture contained 2.67 mL of 50 mM phosphate buffer (pH 7.0), to which 30 μL of enzyme extract and 300 μL of 100 mM H_2O_2 were added to initiate the reaction. The change in absorbance was recorded for 2 minutes at 25°C. CAT activity was determined using the molar extinction coefficient of 39.4 mM^{-1} cm^{-1} for H_2O_2, with one unit of enzyme activity defined as the amount required to decompose 1 μmol of H_2O_2 per minute under the assay conditions.

3.1.6. Proline content determination

The proline content was assayed based on a previous method (Bates et al., 1973) with modification. In short, approximately 0.5 g of leaves were homogenized in 10 mL of 3% sulfosalicylic acid, and the mixture was filtered through Whatman filter paper. The filtrate (2 mL) was incubated with 2 mL acid-ninhydrin and 2 mL of glacial acetic acid at 100oC for 30 min. Then the mixture was extracted with 4 mL toluene, and the absorbance was detected at 520 nm using a TU-1810 ultraviolet spectrophotometer (Purkinje, Beijing, China).

3.1.7. MDA content determination

MDA content was examined using a well-established method reported by Heath and Packer (Heath and Packer 1968) with modification. Briefly, 1 g of leaves was homogenized in Tris–HCl buffer (50 mM, pH 8.0) for 30 s using a BILON-J1500 blender (Waring, New Hartford, Conn., USA). The suspension was filtered through four layers of cotton cloth and then centrifuged for 1 min (200× g). The supernatant was centrifuged at 200× g for 10 min, and the chloroplast precipitate was collected and incubated in a 10 mL tube containing Tris–HCl. An equal aliquot of 0.5% thiobarbituric acid (TBA) in 20% trichloroacetic acid was mixed with the extract. The mixture was heated for 25 min at 95°C. The supernatant was collected by centrifugation and assayed at 530 nm for absorbance.

3.1.8. H_2O_2 content

H_2O_2 was examined according to a previous method reported by Bouaziz et al. (Bouaziz et al. 2015) with modification. Briefly, the leaves (1 g) were digested in 2 mL of 0.1% trichloroacetic acid, followed by centrifugation (12,000 rpm, 15 min). Then, 0.5 mL of supernatant was incubated with 0.5 mL of potassium phosphate (10 mM, pH 7.0) and 1 mL of potassium iodide (1 mol/L). The absorbance was examined at 390 nm.

3.1.9. Total chlorophyll content

Total chlorophyll content in potato leaves was examined using the commercial chlorophyll assay kit according to the manufacturer's instructions (Item No Cat#BC0990; Solarbio, Beijing, China). Fresh potato leaves were collected and washed in distilled water. After draining the surface of the leaves, the midrib of the leaves was removed and cut into pieces. Approximately 100 mg of leaves were weighed and ground thoroughly in 1 mL of water in the dark, which was then entirely transferred into a 10-mL volumetric flask, diluted with water to volume, and mixed. The volumetric flask was maintained in the dark for 3 h. The absorbance of the supernatant was measured at a wavelength of 663 nm and 645 nm using a spectrophotometer model (Perkin Elmer, Shelton, CT, USA).
